# Supplementary material for: Octopamine and tyramine signalling in Aedes aegypti: Molecular characterization and insight into potential physiological roles
Source: PLoS One. 2023 Feb 16;18(2):e0281917. doi: 10.1371/journal.pone.0281917 (PMC9934454; doi:10.1371/journal.pone.0281917)
Supplement: S8 Fig — (DOCX) [file pone.0281917.s008.docx]

**Supplementary figure S8.**


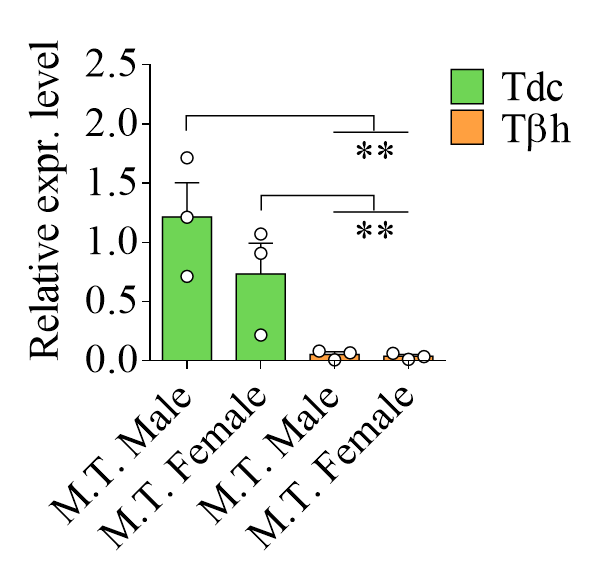


**Supplementary figure S8.** Expression patterns of the enzymes involved in TA and OA biosynthesis: tyrosine decarboxylase (Tdc) and tyramine β-hydroxylase (Tβh). The transcript levels were investigated in Malpighian tubules of adult male and female mosquitoes. Data represent means ± SEM of three biological replicates. Statistical significance is indicated by ** p<0.01 according to one-way ANOVA followed by multiple comparisons Bonferroni post hoc test. Malpighian tubules (MTs).
