# Supplementary material for: Octopamine and tyramine signalling in Aedes aegypti: Molecular characterization and insight into potential physiological roles
Source: PLoS One. 2023 Feb 16;18(2):e0281917. doi: 10.1371/journal.pone.0281917 (PMC9934454; doi:10.1371/journal.pone.0281917)
Supplement: S1 Table — (DOCX) [file pone.0281917.s009.docx]

| Primers used for OA and TA sequences amplification and sequencing | | | | | |
| --- | --- | --- | --- | --- | --- |
| Gene | | Sequence (5’-3’) | | | |
| AaOAα1-R | | Fw: TTCTGGTCCGAGCTGAGATT | | | |
|  |  | Rev: CCTTAAAACCACGCGGAATA | | | |
| AaOAα1-R (ORF) | | Fw: **ATG**AATGCGACCGAGTGC | | | |
|  |  | Rev: **TCA**TCTGCTGTCGGACAGGT | | | |
| AaOAα2-R | | Fw: **ATG**GATTACCCAATGATAGCAAC | | | |
|  |  | Rev: **TCA**CTTGAACAGGATCCGC | | | |
| AaOAβ2-R | | Fw: AATTTCGCAAATTTCGTTCG | | | |
|  |  | Rev: CTGTCACCCGCACAGTTCTA | | | |
| AaOAβ2-R (ORF) | | Fw: **ATG**ATGAATCCTTCCAATGACG | | | |
|  |  | Rev: **TTA**GAGACTCTCGCCGATCTC | | | |
| AaOAβ3-R | | Fw: GAGCCAGTCGGTAGTGGAAG | | | |
|  |  | Rev: TTTTCCAACGACGGATTGAT | | | |
| AaOAβ3-R (ORF) | | Fw: **ATG**GCCCTCAGAGCAATGT | | | |
|  |  | Rev: **CTA**CACGTAGTAGGCGCTGTGT | | | |
| AaTAR1 | | Fw: AATCCGTCACAACCAGAAGG | | | |
|  |  | Rev: ACTCGATTGCTGATGCAGTG | | | |
| AaTAR1 (ORF) | | Fw: **ATG**GCAATTGTATCAGTGATACC | | | |
|  |  | Rev: **CTA**CTGTTTGATTCCGAGCAAT | | | |
| AaTAR2 | | Fw: TTGCGGTACTGTTTGCTCTG | | | |
|  |  | Rev: CATCCGTGTGAAACCACATC | | | |
| AaTAR2 (ORF) | | Fw: **ATG**GAAACTCGCCTCGATG | | | |
|  |  | Rev: **TTA**TCTCCGCATGGACATGT | | | |
| AaTAR3 | | Fw: CTGGATAATCGAACAGCCACT | | | |
|  |  | Rev: AGCCACAGCTTGGTCTTGAT | | | |
| AaTAR3 (ORF) | | Fw: **ATG**GCCAATGAAAGCGG | | | |
|  |  | Rev: **CTA**CGACTTAAAAAACGCCAAAT | | | |
| Primers used for RT-qPCR analysis | | | | | |
| Name | Sequence (3’-5’) | | Size amplicon  (bp) | Regression coefficient (R^2^) | Efficiency (%) |
| AaOAα1-R | Fw: ATCATCGTCGGGTTGTTCAT | | 162 | 0.9908 | 91.02 |
|  | Rev: GGAAAACAGGGCGTAGATCA | |  |  |  |
| AaOAα2-R | Fw: GTGGTGAAACCGCTCAAGTT | | 182 | 0.9999 | 93.98 |
|  | Rev: GGTTCCAGTTCGCTACAAGC | |  |  |  |
| AaOAβ2-R | Fw: GCAACCTGCTCGTCATCATA | | 192 | 0.9984 | 100 |
|  | Rev: CATCCAAACTGTTCCACACG | |  |  |  |
| AaOAβ3-R | Fw: GATTCAGGCAGTCCGGTAAA | | 161 | 0.9998 | 88.18 |
|  | Rev: GGGTTGACACTGTCCTCGTT | |  |  |  |
| AaTAR1 | Fw: GCATCCACGTATGCAAAATG | | 197 | 0.9953 | 95.51 |
|  | Rev: TGGAGAACTGATCAGCAACG | |  |  |  |
| AaTAR2 | Fw: GAGGCTCATCCAGCTTTACG | | 154 | 0.9987 | 93.88 |
|  | Rev: CCCACGAAAGAAGTTGTGGT | |  |  |  |
| AaTAR3 | Fw: GTCAATTTGCAGCTCGTGAA | | 198 | 0.9958 | 106.64 |
|  | Rev: GTCCTTGTCGGTGGAAGTGT | |  |  |  |
| Actin | Fw: CGTTCGTGACATCAAGGAAA | | 175 | 1 | 97.33 |
|  | Rev: GAACGATGGCTGGAAGAGAG | |  |  |  |
| Rps17 | Fw: AAGAAGTGGCCATCATTCCA | | 200 | 0.9946 | 98.31 |
|  | Rev: GGTCTCCGGGTCGACTTC | |  |  |  |
| Rps32 | Fw: CAGTCCGATCGCTATGACAA | | 200 | 0.9874 | 89.10 |
|  | Rev: ATCATCAGCACCTCCAGCTC | |  |  |  |

**Supplementary table T1.** Primers used in this research work. The formula E = (10 ^ ^1/ slope^ -1) * 100 was used to calculate the amplification efficiencies of the genes.
