# Supplementary material for: Octopamine and tyramine signalling in Aedes aegypti: Molecular characterization and insight into potential physiological roles
Source: PLoS One. 2023 Feb 16;18(2):e0281917. doi: 10.1371/journal.pone.0281917 (PMC9934454; doi:10.1371/journal.pone.0281917)
Supplement: Attachment — Submitted filename: Response to reviewers PNTD-D-22-01158.docx [file pone.0281917.s011.docx]

**Response to previous reviewers of manuscript PNTD-D-22-01158 for submission PONE-D-22-34361**

-Are the objectives of the study clearly articulated with a clear testable hypothesis stated?

-Is the study design appropriate to address the stated objectives?

-Is the population clearly described and appropriate for the hypothesis being tested?

-Is the sample size sufficient to ensure adequate power to address the hypothesis being tested?

-Were correct statistical analysis used to support conclusions?

-Are there concerns about ethical or regulatory requirements being met?

**Reviewer #1**: The methods are clear. I would suggest that the authors include some information regarding:
- where the male and female mosquitoes used always mated?

- when collecting antennae, was only the flagellum included or also the 2nd antennal segment (Johnston's organ)?

Reply: The male and female mosquitoes used in the experiments were virgin. When antennae were collected the Johnston’s organ was excluded.

**Reviewer #2**: In the present manuscript, Finetti and others combine bioinformatic approaches and qPCR analyses to examine expression profiles of putative receptors for octopamine and tyramine, as well as their biosynthetic enzymes, in the yellow fever mosquito Aedes aegypti. The methods are appropriate for expression analyses of selected genes.

**Reviewer #3**: There is really no testable hypothesis except exploring if the receptors are expressed in different stages and tissues. The manuscript by Finetti et al. is a descriptive account of the 7 “identified” octopamine and tyramine putative receptors from the Aedes aegypti genome.
The authors have designed primers for the different receptors and performed qRT-PCR experiments to obtain the relative transcript expression using three reference genes in all stages of development and in various tissues, specifically at different points following a blood meal.

There are several areas that require clarification. In my opinion the paper is too descriptive, and it is not a good fit for this particular journal. It is more suitable for an insect molecular biology journal or a general biological interest journal like PLOS One. There is nothing related to neglected and tropical diseases except that the transcriptional expression analyses are in a mosquito vector. The title is misleading because there is nothing in this paper about signaling except pasting the sequences found in NCBI. There are no functional analyses and no pharmacology of any sort.

The sample size of only 5 females per analyses and cDNA synthesis gives a lot of questions about the validity of the qRT-PCR results; only that the receptor is expressed, but the relative quantities may be affected by the small sample size. I understand that they did pools, but mosquito researchers know that there is sufficient variability among mosquitoes including those in laboratory settings that quantitative data on only 5 mosquitoes is of questionable significance. Normally 30 is the accepted minimal number even for typical population structure studies. After pondering the significance of this work, I cannot help than think that this is a preliminary study, in which the authors probably repeated similar work as those annotating the genome to validate the 7 TMs in these sequences (even if done automatically).

Reply: We do not agree with the reviewer about the sample size. There are several works where the gene expression was investigated using five or lesser whole mosquitoes per replicate (Alonso et al., 2019: Ni et al., 2022). Furthermore, we dissected and collected the tissues from at least 25 mosquitoes per replicate, making the expression analyses reproducible and reliable.

We have changed the title.

References:

Alonso, D.P., Campos, M., Troca, H. et al. Gene expression profile of Aedes aegypti females in courtship and mating. Sci Rep 9, 15492 (2019).

Ni, M., Zhao, T., Lv, Hx. et al. Screening for odorant receptor genes expressed in Aedes aegypti involved in host-seeking, blood-feeding and oviposition behaviors. Parasites Vectors 15, 71 (2022).
--------------------

**Results**


-Does the analysis presented match the analysis plan?

-Are the results clearly and completely presented?

-Are the figures (Tables, Images) of sufficient quality for clarity?

**Reviewer #1**: The results are clear and well presented. I only have a few comments:

Line 291: “…associated with smell and taste” and hearing? Georgiades M, Alampounti CA, Somers J, Su M, Ellis D, Bagi J, et al. A novel beta-adrenergic like octopamine receptor modulates the audition of malaria mosquitoes and serves as insecticide target [Internet]. bioRxiv; 2022 [cited 2022 Sep 13]. p. 2022.08.02.502538.

Reply: In the revised manuscript, we added the reference in the text as recommended.

Table 1: No coding DNA sequence shown in first row. Figures: General comment. “α” and “β” are not properly shown in heatmaps. Figure 1 quality is very low; it is difficult to read.
Figures 2-3: legends should be improved. It is not clear what "a,b,c" on the top of bar plots mean and how the statistics were calculated. Also, please briefly mention in the legend how relative expression levels were calculated to facilitate the reading.

Reply: In the revised manuscript, we fixed the heatmaps and the figure 1 quality. Furthermore, we improved all the figure captions.

**Reviewer #2**: Their expression analyses are rigorous and thorough. The results are also consistent among different figures, ensuring reliability of their work. In the heatmap panels in multiple figures, “alpha” and “beta” for putative OA receptors are substituted by “?”. This needs to be corrected.

Reply: We fixed the heatmaps in the revised manuscript.

**Reviewer #3**: 1. The authors indicate that using Drosophila sequences they searched the genome of Aedes aegypti and “Identified” the 7 receptor sequences, but they never indicated how these receptor sequences are currently annotated. My suspicion is that they are all correctly annotated with the exception of the NPY6 early description by the unfortunate error of the Leslie Vosshall’s group.

Reply: This was overlooked by the reviewer since the current receptor annotations were already present in the results (lines 191-197).

2. In the methods and results they talk about the BRAIN but how the brain was dissected is not described. My guess is that what they call brain is the rest of the head without rostrum and antennae, which they analyzed separately. This must be clarified.

Reply: The brain dissections were performed under stereomicroscope in cold PBS opening the head from the eyes by using a micro-dissecting forceps. Use of Brain is correct.

3. The primer efficiency was not disclosed, and the location of the primers is only drawn on the supplementary figures. Considering that these receptors are very similar in sequence the strategy and precautions taken to design the primers should have been discussed or described under materials and methods. Also, they must clearly indicate where in the sequence with respect to the open reading frame the primers are. The reader should not have to search this especially because they are repeating the same nucleotide sequence in two contiguous figures. Just COUNT and indicate exactly for each figure what is the position of the primers and in the same figure indicate where the open reading frame corresponding sequence is. In Fig s1d the authors forgot to indicate that the red square indicates the DRY region. Legend figure S2d should say dots do not dot because there are 3 dots.

Reply: In the revised manuscript, the primer efficiency and their locations were added in the supplementary table 1. Furthermore, we fixed the supplementary figures 1 and 2.

As in S2d, Figure S3b only contains the ORF and the primers are in the middle of it, again they should indicate where they fall if in a transmembrane region or a loop and what searches have been done with the primers to guarantee that there are no non-target sequences also being amplified? For S3b also indicate dots do not dot. Fig S4b only has one primer drawn in red on the figure, what about the other primer? Also, for S5 the same dots do not dot, and the indication of the DRY motif is missing.

Reply: All the supplementary figures were checked and fixed.

The sizes of the amplicons are not indicated, and these could have been easily placed in one of the tables such as T1. Suppl Fig S8 does not add anything and should be deleted.

Reply: The amplicon sizes were added in the supplementary table 1 in the revised manuscript.

The methods are not reproducible: the volume of cDNA used cannot be traced to mosquito or tissue equivalent. At least the total RNA or mRNA equivalent measured before cDNA synthesis should be given. See lines 177 onwards. Also, more detail on how the plates were loaded and which tissues or “treatments” were placed in the same plate, etc.

Reply: The amount of total RNA used to synthesize the cDNA was already present in the material and methods (line 168). We used 384-wells plates to run the RT-qPCR analysis and each replicate was run on the same plate.

In line 270 clarify in the text of the manuscript if the highest amount refers to TAR2, one should not have to look at the figure. In line 238 must add the citation on where the biogenic amines bind the receptor. Same for line 253. Another issue is the poor literature review. Peter Piermarini has worked on the Aedes aegypti crop and octopamine contracts the crop. Why not checking which receptors are in the crop in addition to the midgut?

Reply: The text was improved as suggested in the revised manuscript.

Editing errors/ suggestions

Line 65 yellow fever viruses, which cause debilitating.

Line 82 Year for Ngai et al?

Line 355 I would not write accumulation because that may imply slow turnover of the transcript. Just say that relatively more was detected.

The reference of Hixson et al in line 528 is missing from the reference list. I suggest the authors use ENDNOTE or similar reference database like ZOTERO (is free).

Reply: All the references were checked, and the suggestions fixed in the revised manuscript.

--------------------

**Conclusions**


-Are the conclusions supported by the data presented?

-Are the limitations of analysis clearly described?

-Do the authors discuss how these data can be helpful to advance our understanding of the topic under study?

-Is public health relevance addressed?

**Reviewer #1**: The authors provide an interesting overview and discussion of their findings in light of the available literature.

**Reviewer #2**: Results of their detailed analyses are expected to inform future efforts to target these biogenic amines signaling pathways for controlling this disease vector species.

**Reviewer #3**: In the discussion the authors try to indicate why they analyzed each tissue but very few conclusions can be made from such a qRT-PCR study.

--------------------

**Editorial and Data Presentation Modifications?**

Use this section for editorial suggestions as well as relatively minor modifications of existing data that would enhance clarity. If the only modifications needed are minor and/or editorial, you may wish to recommend “Minor Revision” or “Accept”.

**Reviewer #1**: (No Response)

**Reviewer #2**: 1) Lines 32-35: If the blood meal influences receptor expression patterns, it is likely that they are involved in post-feeding events such as digestion, excretion, or reproduction, rather than feeding per se. 2) Figure 2G indicates that OAbeta3-R is relatively highly expressed in females as compared to other putative OA receptors, whereas such pattern is not observed in tissue-specific expression analyses in Figure 3. It may be informative to discuss which tissue potentially contributes to its high expression in females.

Reply: Since we investigated the expression profile of the TA and OA receptors in antennae+rostrum, brain, Malpighian tubules, midgut and ovaries, the high OAβ3-R expression observed in the whole *A. aegypti* female might be related to other tissues, such as fat body, hindgut, salivary glands which were not examined in this study.

**Reviewer #3**: (No Response)

--------------------
**Summary and General Comments**

Use this section to provide overall comments, discuss strengths/weaknesses of the study, novelty, significance, general execution, and scholarship. You may also include additional comments for the author, including concerns about dual publication, research ethics, or publication ethics. If requesting major revision, please articulate the new experiments that are needed.

**Reviewer #1**: This study is relevant and timely. It presents a detailed analysis of the identity and expression of octopamine and tyramine receptors in one of the most important disease-transmitting mosquitoes, Aedes aegypti. The authors investigate the effects of development, tissue, and blood-feeding state on the expression levels of different receptors. Octopamine receptors are promising targets for the development of novel insecticides, so this study is not only relevant from a vector biology perspective, but also can inform the development of control tools based on targeting tyraminergic or octopaminergic pathways.

**Reviewer #2**: The strength of this manuscript is thoroughness of their expression analyses. The results are also consistent among different figures, ensuring reliability of their work. On the other hand, the weakness of their work is that they only performed expression analyses, and therefore they fall short of their claim that they “characterized” (as they claim in the title) octopamine and tyramine signaling in Aedes. The GPCRs they analyzed here are just “putative” octopamine/tyramine receptors without functional characterization in heterologous systems, such as cultured cells or Xenopus oocytes. Often times, these biogenic amine receptors have varying levels of cross-reactivity for different ligands. Therefore, calling them either “OA” or ‘TA” receptors without appropriate functional analyses can potentially mislead future studies. Numerous loss-of-function analysis tools (such as CRISPR/Cas and RNAi) are also available in Aedes, which allows them to test their claim that these receptors can be novel targets for Aedes control. Without these additional experiments, their claim that they characterized these biogenic amines signaling pathways in Aedes is not well justified, and thus their work does not meet the criteria for publication in PNTD.

**Reviewer #3**: I included my comments above under the results section but please read them above.
