# Supplementary material for: Octopamine and tyramine signalling in Aedes aegypti: Molecular characterization and insight into potential physiological roles
Source: PLoS One. 2023 Feb 16;18(2):e0281917. doi: 10.1371/journal.pone.0281917 (PMC9934454; doi:10.1371/journal.pone.0281917)
Supplement: Attachment — Submitted filename: My main concern.docx [file pone.0281917.s013.docx]

The manuscript gives a good contribution in the identification and molecular characterization of four OA and three TA receptors in *A. aegypti* and their putative physiological roles based on transcript expression profile analysis will be useful for better understanding complex mechanisms such as reproduction and osmoregulation in this important vector of several human diseases. However, I have concerns about the methods, results and discusssion of the ms that I believe need to be addressed in order to improve its clarity. Their approach is interesting but it has some flaws that make this version unacceptable for publication.

My main concern:

1- Line 122: Why did you choose 12/12 instead of 8/16 D/L cycle?
2- Line 301: I would suggest to put subheading as a sentence instead of question form.
3- Line 303: What do authors mean with (Female A. aegypti require a blood meal for egg production and it is also true that this process imposes some stress on the insect?)
4- Line 316: Unfed means starved or sugar-fed? First, because it is well known that starvation stimulate stess hormone, octopamine, the counterpart of adrenaline in vertebrates and consequently alter the expression level of the corresponding receptors. Second, did authors compare their control at the same time points?
5- Heat maps included in figure (4-7) did not add more information especially no significant difference among chosen time points are found.
6- Line 422: The expression level of *Tβh* in males is highly upregulated, however, tests (the only exclusive organ to males among all chosen organs) show very low expression level, from where this difference appear? How?
7- Lines 484-486: According to Fig.8, this upregulation was not statistically significant, you should not magnify your results if they are not statistically significant. I suggest to focus on the most important and statistically significant data in the whole ms.
8- Line 501: Did you compare statistically the expression level of *tdc* with the expression level of *Tβh*? If not, how did you conclude that *tdc* was more highly expressed than *Tβh* in MTs?
9- Line 517-518: Regarding the sentence (and has not been highly investigated in reproductive organs but was shown to be present in D. melanogaster female reproductive organs (El-Kholy et al., 2015).

I suggest to delete this sentence because El-Kholy et al (2015) detect very weak TyRII expression level in male and female reproductive system (almost nothing) but the strongest expression of this receptor was detected in intestine and MTs.

10- Lines (526-530): Regarding the sentence (Surprisingly, blood feeding did not affect the expression profile of TA and OA receptors in the brain of female mosquitoes. On the other hand, transcripts encoding the enzymes responsible for the synthesis of TA and OA, respectively Tdc and Tβh, showed dynamic profiles, with peaks in the first hours after blood feeding, suggesting that blood intake induces a strong synthesis of both TA and OA), This means that in female mosquitoes, blood meal induce more OA and TA to be synthetized but without any consequent change in their related receptors, please explain.

11- Line 505: Revise the written mistakes in all references in the text for example replace (El-Kholi et al. 2015) with (El-Kholy et al. 2015).
